# Three-Dimensional Left Atrial Geometry in Atrial Fibrillation: Imaging Biomarkers, Substrate Phenotyping, and Ablation Outcome Prediction

**DOI:** 10.3390/diagnostics16142255

**Published:** 2026-07-19

**Authors:** Paschalis Karakasis, Panagiotis Theofilis, Panagiotis Stachteas, Konstantinos Grigoriou, Panagiotis Iliakis, Athina Nasoufidou, Panayotis K. Vlachakis, Nikolaos Ktenopoulos, Anastasios Apostolos, Theodoros Karamitsos, Antonios P. Antoniadis, Nikolaos Fragakis

**Affiliations:** 1 Second Department of Cardiology, Hippokration General Hospital, Aristotle University of Thessaloniki, Konstantinoupoleos 49, 54642 Thessaloniki, Greece; 2 First Cardiology Department, School of Medicine, Hippokration General Hospital, National and Kapodistrian University of Athens, 11527 Athens, Greece; 3 Department of Pharmacology, University of Athens, 75 Mikras Asias Avenue, 11527 Goudi, Greece; 4 Department of Medicine, Division of Cardiology, Angiology and Internal Emergency Medicine, Ruhr University Bochum, Knappschaft Kliniken University Hospital Bochum, 44892 Bochum, Germany; 5 Department of Cardiology, Guy’s and St Thomas’ NHS Foundation Trust, Harefield Hospital, London UB9 6JH, UK; 6 Third Department of Cardiology, Hippokration General Hospital, Aristotle University of Thessaloniki, 54642 Thessaloniki, Greece

**Keywords:** atrial fibrillation, left atrial remodeling, three-dimensional left atrial geometry, atrial cardiomyopathy, catheter ablation, pulsed field ablation, imaging biomarkers, substrate phenotyping, left atrial sphericity, artificial intelligence

## Abstract

Assessment of left atrial remodeling in atrial fibrillation (AF) has traditionally relied on anteroposterior diameter, left atrial volume (LAV), and indexed left atrial volume (LAVI). Although these measures remain clinically useful, they reduce a complex, asymmetric, and anatomically constrained chamber to scalar descriptors and therefore cannot fully capture the spatial substrate that underlies AF persistence, thromboembolic risk, or arrhythmia recurrence after catheter ablation. Three-dimensional left atrial reconstruction provides a more refined framework by preserving chamber shape, regional deformation, pulmonary vein (PV) orientation, left atrial appendage (LAA) geometry, posterior wall and roof configuration, left lateral ridge anatomy, wall-thickness heterogeneity, and computational surface features. In this review, we examine how three-dimensional left atrial geometry can extend conventional remodeling assessment from measurement of atrial size toward imaging-based substrate characterization. We discuss the relative strengths and limitations of computed tomography (CT), cardiovascular magnetic resonance (CMR), three-dimensional echocardiography, and electroanatomic mapping (EAM), and summarize key geometry-derived metrics, including LAV, LAVI, left atrial sphericity, asymmetry index, atrial eccentricity index, PV anatomy, LAA morphology, posterior wall geometry, wall thickness, radiomics, and artificial intelligence (AI)-derived shape descriptors. We further synthesize evidence linking geometric remodeling with atrial cardiomyopathy, mechanical dysfunction, fibrosis, low-voltage substrate, and catheter ablation outcomes. The clinical relevance of three-dimensional left atrial geometry may be further redefined by pulsed field ablation (PFA), whose non-thermal lesion biology and tissue selectivity may modify predictors of recurrence established in radiofrequency and cryoballoon cohorts. Finally, we outline the need for standardized segmentation, harmonized metric definitions, prospective multicenter validation, and integration with AI, digital twin modeling, biomarkers, EAM data, and wearable-derived AF burden. Three-dimensional left atrial geometry is not yet a standalone determinant of ablation strategy, but it may become a central component of individualized atrial phenotyping and rhythm-control decision-making.

## 1. Introduction

Atrial fibrillation (AF) is the most common sustained cardiac arrhythmia and represents a growing global public health challenge, driven by population aging, cardiometabolic disease, heart failure, and improved rhythm detection [1,2]. Contemporary management has shifted from a narrow rhythm-versus-rate paradigm toward integrated, longitudinal care that combines stroke prevention, risk-factor modification, symptom control, and early rhythm-control strategies in appropriately selected patients [3,4,5]. This transition reflects a broader recognition that AF is not merely an episodic electrical disorder, but the clinical manifestation of a progressive atrial disease process shaped by structural, mechanical, metabolic, inflammatory, autonomic, and electrophysiological remodeling [6].

Catheter ablation has transformed rhythm-control therapy since the recognition of the pulmonary veins (PV) as dominant triggers of paroxysmal AF [7]. Nevertheless, outcomes remain heterogeneous, particularly in persistent AF and in patients with advanced comorbidity or established atrial substrate. Seminal electroanatomic and histological studies have shown that pre-existing atrial scar, fibrosis, tissue disarray, and structural remodeling are strongly linked to procedural failure and arrhythmia maintenance [8,9]. Thus, the central clinical challenge is no longer only how to isolate the pulmonary veins, but how to identify the patient-specific atrial substrate that determines whether pulmonary vein isolation alone is sufficient or whether recurrence reflects more diffuse atrial cardiomyopathy (AtCM) [10,11].

Conventional assessment of left atrial (LA) remodeling has relied predominantly on linear dimensions and volumetric indices [12]. Although clinically useful, these measures compress a complex, asymmetric, and regionally heterogeneous chamber into scalar parameters and therefore provide limited information on the spatial architecture of atrial disease [13]. Three-dimensional LA reconstruction offers a more anatomically informative approach by preserving chamber shape, regional deformation, pulmonary venous anatomy, appendage configuration, posterior wall geometry, wall-thickness heterogeneity, and spatial relationships with adjacent structures [14,15]. These features may represent the macroscopic anatomical expression of AtCM and may improve risk stratification beyond conventional size-based assessment [16].

**Figure 1:**
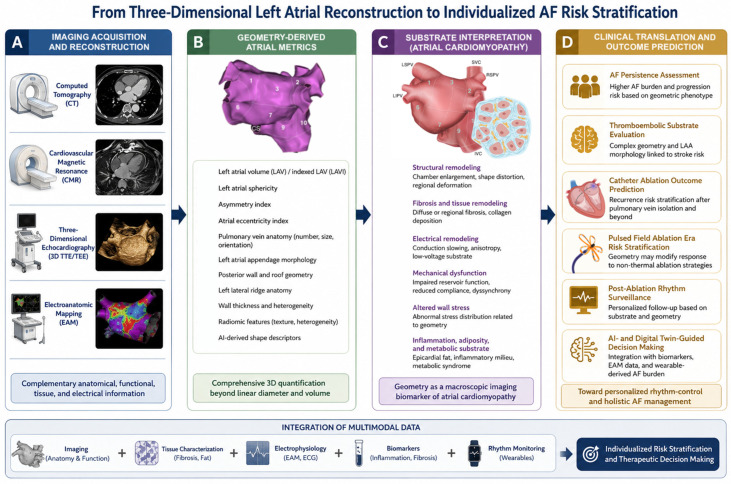
From three-dimensional left atrial reconstruction to individualized atrial fibrillation risk stratification. Multimodality imaging and mapping platforms provide complementary inputs for three-dimensional left atrial reconstruction. Quantitative geometric analysis extends conventional assessment beyond linear diameter and volume by capturing global shape, regional deformation, pulmonary venous anatomy, appendage morphology, posterior wall configuration, wall-thickness heterogeneity, and computational surface features. These metrics may act as macroscopic imaging biomarkers of atrial cardiomyopathy and can be integrated with functional, fibrotic, electrical, biomarker, and rhythm-monitoring data to refine atrial fibrillation risk stratification, catheter ablation outcome prediction, and post-ablation surveillance.

The need for refined geometric phenotyping is particularly relevant in the current era of pulsed field ablation (PFA). Because PFA differs fundamentally from radiofrequency and cryoballoon ablation in lesion biology, tissue selectivity, and collateral injury profile, predictors derived from thermal ablation cohorts may not be directly transferable to this new energy modality [17,18]. In parallel, advances in cardiac CT, CMR, electroanatomic mapping (EAM), radiomics, artificial intelligence, and digital twin modeling now make it possible to move from descriptive imaging toward patient-specific atrial phenotyping (Figure 1). This review synthesizes the role of 3D LA geometry in AF evaluation, with emphasis on imaging platforms, quantitative geometric metrics, AtCM, ablation outcomes, PFA-era implications, and future integration with multimodal and computational approaches.

## 2. From LA Diameter to 3D Morphology: Limitations of Conventional Assessment

### 2.1. Limitations of Anteroposterior LA Diameter

LA size has traditionally been assessed using the anteroposterior LA diameter because it is simple, reproducible, and widely available during routine echocardiography [19]. This metric retains prognostic value in AF, and larger LA diameter has been associated with recurrence after rhythm-control interventions, including catheter ablation [20]. However, its biological and anatomical fidelity is limited. A single linear dimension reduces a geometrically complex chamber to one axis and implicitly assumes that LA enlargement proceeds symmetrically. This assumption is problematic because AF-related remodeling is spatially heterogeneous and may preferentially involve the superior-inferior, mediolateral, anterior, posterior, roof, or appendage-related components of the atrium. Consequently, LA diameter may remain only modestly abnormal despite substantial volumetric or regional remodeling, particularly when dilatation occurs outside the anteroposterior plane [21].

Guideline-based chamber quantification has therefore moved away from isolated linear measurements and recommends volumetric assessment for LA enlargement whenever feasible [19]. The rationale is not merely technical. LA diameter is vulnerable to acoustic-window limitations, foreshortening, loading conditions, and distortion of anteroposterior chamber geometry by adjacent structures, particularly the aortic root/descending aorta and posterior thoracic constraints [22]. More importantly, it cannot capture compartmental remodeling, pulmonary venous antral expansion, posterior wall configuration, or left atrial appendage (LAA) morphology. Thus, although LA diameter may function as a crude marker of cumulative atrial burden, it is insufficient as a phenotype of AtCM.

### 2.2. LAV and LAVI: Useful but Incomplete

Left atrial volume (LAV) and indexed left atrial volume (LAVI) provide a more robust estimate of LA remodeling than diameter or area and have consistently outperformed linear measurements for cardiovascular risk prediction [12,23]. In AF ablation cohorts, larger LAV and LAVI have been associated with higher recurrence risk, and meta-analytic evidence supports their value as preprocedural predictors of post-ablation failure [24]. This has made LAVI a central structural marker in AF evaluation, reflecting the cumulative effect of pressure overload, volume overload, impaired LV filling, mitral valve disease, obesity, hypertension, and sustained arrhythmic burden.

Nevertheless, LAV remains a scalar descriptor. It quantifies chamber size but not chamber architecture. Two patients may have identical LAV but profoundly different LA phenotypes: one may have a relatively symmetric, spherical atrium; another may exhibit eccentric elongation, preferential anterior expansion, posterior wall flattening, roof enlargement, PV ostial distortion, or a large, complex LAA. These differences may carry distinct implications for wall stress, conduction anisotropy, regional fibrosis, catheter contact, lesion deployment, and arrhythmia recurrence. Moreover, 2D echocardiographic LAV may underestimate true LA volume compared with CMR or CT because of foreshortening and incomplete endocardial border definition [21]. Three-dimensional echocardiography reduces some of these assumptions and improves volumetric characterization, but CT and CMR remain more suitable when detailed surface geometry, PV anatomy, and LAA morphology are required [25].

Accordingly, LAV and LAVI should be viewed as necessary but not sufficient markers. They identify the magnitude of atrial enlargement but do not explain how the atrium has remodeled. This distinction is critical in AF, where the arrhythmogenic substrate is not determined only by total chamber size but also by the spatial distribution of structural deformation [23].

### 2.3. Why Atrial Remodeling Is Not Spherical or Uniform

AF-related left atrial (LA) remodeling is not a passive, homogeneous expansion of a spherical chamber; rather, it is a spatially heterogeneous process characterized by asymmetric enlargement, regional deformation, and progressive changes in chamber shape [26]. The LA is anatomically constrained by neighboring structures and contains functionally distinct regions, including the posterior wall, PV antra, roof, mitral isthmus, septum, vestibule, and LAA. These components remodel unevenly under chronic pressure, volume, metabolic, and inflammatory stress [27,28]. Early echocardiographic work demonstrated that LA dilatation may assume a trapezoidal pattern, with disproportionate basal enlargement relative to apical dimensions, highlighting the inadequacy of assuming uniform enlargement [29].

More recent 3D imaging studies have reinforced this concept. CT-derived asymmetry index (ASI) demonstrated that AF is associated with asymmetric LA remodeling, particularly preferential anterior expansion, and that this asymmetry differs across AF phenotypes and may predict ablation outcomes [13,30]. Similarly, left atrial sphericity (LASP) was introduced to quantify the transition from a discoid or irregular LA configuration toward a more spherical chamber, with higher sphericity associated with persistent AF and recurrence after ablation in several cohorts [31,32,33]. However, not all studies have shown identical results, suggesting that the prognostic meaning of LA geometry may depend on AF phenotype, imaging modality, segmentation method, rhythm at acquisition, and whether PVs or LAA are included in the reconstructed model [34].

Beyond global shape, regional anatomy also matters. PV number, ostial dimensions, common trunks, and antral orientation vary considerably between patients and directly influence ablation planning [35,36,37,38,39]. LAA morphology and volume may contribute to thromboembolic risk and may also reflect broader atrial remodeling. The posterior LA wall, in turn, is anatomically and electrophysiologically important because of its proximity to the PVs, esophagus, autonomic inputs, and potential non-PV substrate. None of these features can be adequately inferred from LA diameter or LAV alone.

### 2.4. Geometric Remodeling as a Substrate Marker

The shift from LA diameter to 3D morphology reflects a broader conceptual transition: from measuring atrial size to phenotyping atrial substrate. Contemporary AtCM frameworks emphasize that AF is embedded within a progressive atrial disease process involving structural, electrical, contractile, autonomic, and molecular remodeling [40]. LA geometry may represent a macroscopic readout of these processes. Shape deformation can integrate the long-term effects of wall stress, fibrosis, chamber stiffness, impaired reservoir function, altered activation, and regional conduction heterogeneity.

This interpretation is supported by studies showing that 3D LA shape metrics can predict rhythm-control outcomes independently of conventional clinical variables and, in some analyses, beyond LAV [41]. Particle-based modeling, statistical shape analysis, and CMR/CT-derived geometric indices have identified specific global and regional shape patterns associated with AF recurrence after ablation [14,31,32,42]. These findings suggest that geometry is not merely a descriptive anatomical feature but a clinically meaningful substrate marker.

Therefore, 3D LA reconstruction provides a more biologically coherent framework for AF evaluation than conventional measurements [26]. It preserves volumetric information while adding shape, regionality, and anatomical context. In practical terms, it enables clinicians and researchers to distinguish patients with similar atrial size but different remodeling phenotypes—an essential step toward individualized risk stratification, substrate characterization, and geometry-informed ablation planning.

## 3. Imaging Platforms for 3D LA Reconstruction

Three-dimensional LA reconstruction can be performed using several imaging and mapping platforms, each capturing a different dimension of atrial remodeling (Table 1). CT provides high-resolution anatomical definition; CMR combines anatomy with tissue characterization and functional assessment; 3D echocardiography offers accessible volumetric and mechanical evaluation; and EAM provides invasive, procedure-specific electrical and anatomical information. These modalities should therefore not be viewed as interchangeable, but as complementary tools positioned along a continuum from preprocedural anatomical phenotyping to intraprocedural substrate assessment.

### 3.1. Cardiac CT

Cardiac CT is the most practical platform for high-fidelity preprocedural anatomical modeling of the LA [56]. Its principal strength is isotropic spatial resolution, which permits accurate segmentation of the LA body, PV ostia, posterior wall, mitral isthmus, left lateral ridge, and LAA. Early multidetector CT studies established its value as a “road map” before AF ablation by demonstrating reliable depiction of PV number, ostial dimensions, common trunks, accessory veins, and LA-PV anatomical variants [43,44,57,58]. This is clinically important because PV anatomy is highly variable and may influence transseptal orientation, catheter stability, device positioning, lesion deployment, and the risk of incomplete antral isolation [58].

Compared with echocardiography, CT is less dependent on acoustic windows and geometric assumptions, and compared with EAM-derived shell geometry, it provides a complete, preacquired anatomical surface rather than a catheter-sampled approximation. Integration of CT-derived LA/PV anatomy into EAM systems has been shown to be technically feasible and accurate, with early studies reporting improved anatomical navigation and reduced registration error when surface-based matching was used [59,60]. Subsequent randomized data suggested that CT image integration may facilitate procedural planning, although its incremental effect on long-term rhythm outcomes is less consistent and likely depends on operator workflow, mapping system, lesion strategy, and baseline anatomical complexity [45].

For geometric phenotyping, CT is particularly attractive because it enables reproducible extraction of global and regional shape indices, including LAV, LAVI, LASP, ASI, atrial eccentricity index (AEI), PV ostial dimensions, LAA morphology, and surface curvature. Unlike single-plane diameter or biplane volumetry, CT preserves the spatial relationships among the LA body, PV antra, posterior wall, LAA, and surrounding structures. This makes CT especially suitable for anatomical digital twinning [61], where the objective is not simply to measure atrial size but to construct a patient-specific representation of atrial architecture. Limitations include radiation exposure, iodinated contrast use, dependence on acquisition phase and rhythm regularity, and potential variability introduced by segmentation thresholds and the inclusion or exclusion of PVs and LAA [62]. These limitations can be mitigated by using clinically indicated preprocedural datasets, ECG-gated reconstruction, thin-slice protocols, and standardized segmentation workflows.

### 3.2. CMR

CMR provides a broader substrate phenotype than CT because it can combine LA anatomy, chamber function, strain, flow, and tissue characterization without ionizing radiation [63]. Magnetic resonance angiography was among the first modalities used to characterize PV anatomy in patients undergoing AF ablation, demonstrating that AF patients have larger PVs than controls and that preprocedural imaging can define PV number, size, and shape [46]. CMR is also uniquely positioned for LGE-based assessment of LA fibrosis and ablation scar. In the DECAAF study, pre-ablation atrial fibrosis quantified by delayed-enhancement MRI was independently associated with recurrent arrhythmia after catheter ablation, supporting the concept that imaging can identify the structural substrate underlying rhythm-control failure [47].

CMR has also been used for advanced shape analysis. Statistical shape modeling and particle-based modeling studies have shown that LA shape carries prognostic information beyond conventional clinical variables, and that shape combined with fibrosis may improve risk stratification after ablation [14,42]. These data are highly relevant because they support a transition from scalar measures of LA enlargement to integrated anatomical-substrate phenotyping.

However, CMR remains less practical than CT for routine pre-ablation anatomical modeling in many centers. LA wall thickness is small, LGE acquisition is technically demanding, segmentation is time-consuming, image quality may be affected by arrhythmia and respiratory motion, and inter-center reproducibility remains challenging. The DECAAF II randomized trial further emphasized that prognostic substrate detection does not automatically translate into therapeutic benefit, as MRI-guided fibrosis ablation added to PVI did not significantly reduce atrial arrhythmia recurrence compared with conventional catheter ablation [48]. Thus, CMR is highly valuable for mechanistic substrate characterization, but CT remains more pragmatic when the primary aim is robust, high-resolution anatomical reconstruction before ablation.

### 3.3. Three-Dimensional Echocardiography

3D echocardiography improves substantially on conventional 2D LA assessment by reducing geometric assumptions and enabling real-time volumetric and functional evaluation. Multicenter validation against CMR has shown that 3D echocardiography provides more accurate and reproducible LAV measurements than 2D methods, with fewer patients misclassified as having normal LA size [49]. Similarly, validation against multidetector CT demonstrated close correlation between 3D echocardiographic and CT-derived LAV, although 3D echocardiography still modestly underestimated LA volume [50]. In AF ablation cohorts, real-time 3D echocardiography has been used to assess LA volume and function before and after ablation, with impaired reverse remodeling and persistent LA dysfunction associated with arrhythmia recurrence [51].

The main advantage of 3D echocardiography is clinical accessibility. It can be repeated serially, does not require radiation or contrast, and captures dynamic LA function rather than static anatomy alone. Three-dimensional transesophageal echocardiography can also visualize PV ostia and LAA anatomy and has been explored as an alternative preprocedural tool before ablation [52]. Nevertheless, echocardiographic 3D reconstruction remains limited by spatial resolution, acoustic-window dependency, operator variability, incomplete visualization of PVs and posterior LA structures, and lower suitability for detailed surface-based geometric indices. Consequently, 3D echocardiography is best regarded as a functional and volumetric complement to CT or CMR rather than a definitive platform for high-resolution anatomical digital twinning.

### 3.4. Electroanatomic Mapping

EAM differs fundamentally from CT, CMR, and echocardiography because it is acquired during the ablation procedure and combines geometry with local electrical information [64,65,66]. Its value lies not in preprocedural anatomical screening but in substrate interrogation: voltage amplitude, activation timing, conduction velocity, fractionation, and regional low-voltage areas can be spatially projected onto a reconstructed LA shell. Studies using voltage-guided approaches have shown that low-voltage substrate is common in AF, particularly in persistent disease, and may predict recurrence after catheter ablation [53,54,55]. High-density mapping catheters further improve spatial sampling and may reduce some limitations of conventional bipolar voltage mapping [67].

However, EAM-derived geometry is not a true imaging reconstruction. It is dependent on catheter contact, point density, respiratory and cardiac motion, chamber deformation, rhythm during acquisition, interpolation algorithms, and operator sampling [64]. Voltage thresholds also vary according to mapping rhythm, electrode size, interelectrode spacing, wavefront direction, contact quality, and atrial wall thickness. For these reasons, EAM is best conceptualized as an intraprocedural functional overlay rather than a substitute for preprocedural CT or CMR anatomy. Its most powerful role is therefore integrative: CT or CMR can provide the anatomical scaffold, while EAM adds electrical substrate information that may clarify the functional consequences of geometric remodeling.

### 3.5. Integration of Imaging-Derived Geometry with Functional Electrophysiological Substrate

An important translational direction is the co-registration of imaging-derived anatomy with the functional electrophysiological substrate. CT can provide patient-specific chamber geometry and regional wall-thickness information, whereas LGE-CMR can characterize fibrotic remodeling; high-density EAM adds spatially resolved information on bipolar and unipolar voltage, local activation timing, conduction velocity, electrogram duration and fractionation, and wavefront-dependent conduction behavior. Emerging artificial intelligence–enabled intracardiac echocardiography (ICE)–EAM platforms, such as the CARTOSOUND SONATA Module, may further facilitate this integration by generating real-time three-dimensional chamber reconstructions and automated anatomical labeling directly from intraprocedural ICE data, thereby extending its role beyond procedural guidance toward dynamic anatomical mapping [68]. Recent CT–EAM data further support a direct structure–function interface, with regional LA wall thickness associated with bipolar voltage, low-voltage substrate, conduction velocity, and complex electrograms [69]. Nevertheless, structural and electrical abnormalities are biologically related but not interchangeable. Combined CMR–EAM studies have associated LGE with lower voltage and slower conduction, whereas studies using high-definition mapping and a common anatomical reference frame have demonstrated only partial spatial concordance among LGE, low-voltage substrate, and slow-conduction regions, with important regional discrepancies, particularly at the posterior LA [64,70,71,72,73]. Moreover, high-density mapping indicates that the apparent atrial substrate is dynamic and may vary according to activation rate and wavefront direction, emphasizing the importance of standardized rhythm and pacing conditions when functional substrate is characterized [74].

A useful conceptual parallel is provided by the ADAS 3D workflow developed in the ventricular tachycardia field. In this setting, LGE-CMR can be transformed into multilayer three-dimensional scar models that distinguish dense scar core from border zone and identify corridors of heterogeneous tissue potentially corresponding to conducting channels [75]. Subsequent studies have linked imaging-derived channel characteristics with arrhythmogenicity and demonstrated spatial relationships between CMR-defined conducting channels and EAM deceleration zones identified using isochronal late-activation mapping [76,77]. In the atrium, ADAS 3D-LA and related post-processing platforms can similarly generate patient-specific three-dimensional fibrosis maps; however, the estimated extent and distribution of LA fibrosis remain sensitive to the post-processing method and thresholding strategy used [78,79].

An AF-specific extension of this ventricular paradigm could therefore integrate CT-derived geometry and wall thickness, CMR-defined fibrosis, and high-density EAM measures of voltage, activation, conduction velocity, and functional slowing within a common anatomical reference frame. Conceptually, such multimodal fusion could help distinguish electrically inactive dense scar from viable but slow-conducting heterogeneous tissue, identify regions where structural and functional abnormalities converge, and provide a more mechanistic basis for atrial substrate phenotyping and selective adjunctive ablation. However, this concept remains hypothesis-generating. The thin atrial wall, cardiac and respiratory motion, registration error, rhythm and wavefront dependence of voltage and conduction measurements, and incomplete spatial concordance between imaging and electrical abnormalities currently limit the direct transfer of ventricular workflows to AF. Prospective multicenter studies are therefore needed to establish whether multimodal image–EAM integration improves substrate classification, target identification, patient selection, or clinical outcomes beyond PVI alone.

## 4. Quantitative 3D Atrial Geometry Metrics

Quantitative three-dimensional atrial geometry extends atrial phenotyping beyond linear and volumetric assessment by capturing chamber size, global shape, regional asymmetry, pulmonary venous anatomy, appendage configuration, posterior wall morphology, wall thickness, and computational shape signatures. This is particularly relevant in AF, where patients with similar LA volume may exhibit distinct patterns of sphericity, anterior expansion, eccentricity, pulmonary vein orientation, left atrial appendage geometry, posterior wall deformation, and wall-thickness heterogeneity. These features may influence substrate complexity, procedural performance, lesion durability, and post-ablation recurrence.

The principal 3D atrial geometry metrics, their technical definitions, biological interpretation, and clinical relevance across AF phenotyping and ablation outcome prediction are summarized in Table 2.

### 4.1. LAV and LAVI: Conventional Volumetric Burden

LAV and LAVI remain the most established 3D measures of LA structural remodeling. They quantify the cumulative volumetric burden imposed by chronic pressure overload, volume overload, impaired LV filling, mitral valve disease, obesity, hypertension, metabolic dysfunction, and AF itself [26]. Compared with anteroposterior LA diameter, LAV more accurately reflects chamber enlargement because it incorporates multidirectional expansion. Accordingly, LAV and LAVI have been consistently associated with AF persistence, rhythm-control failure, and recurrence after catheter ablation [24,49,51,80,81,127]. In a meta-analysis of patients undergoing AF ablation, larger preprocedural LAV was associated with increased risk of recurrence, supporting volumetric burden as a clinically useful predictor [24]. Primary imaging studies have similarly suggested that LAV may be more informative than AF type alone for predicting long-term ablation success [80], while CMR-based analyses have linked LAV with sphericity, fibrosis, and recurrence [81].

However, LAV is a scalar metric. It defines how large the atrium is, but not how it has remodeled. A spherical LA, an elongated LA, and an asymmetrically expanded LA may have comparable volumes but different wall stress profiles, PV geometry, posterior wall dimensions, LAA orientation, and arrhythmogenic substrate. Three-dimensional echocardiographic studies have improved volume estimation compared with 2D methods and have shown that minimum LA volume and LA functional indices may predict recurrence after ablation [50,82,83]. Nevertheless, even accurately measured LAV cannot capture regional geometry. Thus, LAV/LAVI should be interpreted as foundational but incomplete markers: they define the magnitude of atrial remodeling, whereas shape-based indices define its architecture.

### 4.2. LASP: Spherical Remodeling and Global Shape Transformation

LASP quantifies global LA shape remodeling by assessing the degree to which the LA approximates a spherical configuration or, depending on the formula used, deviates from an idealized sphere. Several approaches have been used. Some studies calculate sphericity from the relationship between measured LA volume and the theoretical volume of a sphere, whereas others use surface-based or point-cloud methods based on distances from the LA endocardial surface to the center of mass or to a best-fit sphere [31,32,33,42,81,84,85,86,87]. Because the directionality of the metric varies across formulas, studies should explicitly state whether higher values indicate greater sphericity or greater deviation from a sphere.

The biological rationale for LASP is that spherical remodeling reflects advanced chamber adaptation to chronic hemodynamic and arrhythmic stress. A spherical chamber minimizes surface area relative to volume and may therefore represent a geometric response to sustained wall stress [128]. In AF, increased sphericity may also reflect loss of normal LA regional architecture, PV antral distortion, progressive AtCM, impaired mechanical function, and a more extensive arrhythmogenic substrate. Bisbal et al. first introduced LA sphericity as a marker of atrial remodeling and demonstrated its association with recurrence after AF ablation [31]. The multicenter LAGO-AF study subsequently confirmed the prognostic value of LA geometry, showing that LA sphericity and AF phenotype were among the strongest predictors of ablation outcome [33]. Nakamori et al. demonstrated incremental prognostic value of CMR-derived geometric remodeling after PVI, including the dynamic relationship between LA shape and post-ablation recurrence [32]. Bieging et al. further showed that particle-based LA shape analysis predicted recurrence and improved risk stratification when integrated with fibrosis quantification [42].

LASP has also been evaluated beyond catheter ablation. Osmanagic et al. showed that an echocardiographic LASI predicted early AF recurrence after direct-current cardioversion, supporting its relevance as a rhythm-control marker rather than a purely ablation-specific metric [85]. Shi et al. similarly reported that TTE-derived LASI predicted recurrence after RFCA, particularly in patients with mild-to-moderate LA enlargement [86]. Mechanistic support comes from Hopman et al., who demonstrated an association between LA sphericity and impaired LA strain/strain-rate parameters on CMR feature tracking, linking global shape remodeling with atrial mechanical dysfunction [87].

The thromboembolic relevance of LASP has also been explored. Bisbal et al. reported that LA geometry improved risk prediction for thromboembolic events in AF beyond conventional clinical assessment [84]. CT-based analyses in ischemic stroke cohorts, including ASSAM-related work, have suggested that LA sphericity may reflect atrial myopathy in patients with prior stroke, while more recent work in embolic stroke of undetermined source has evaluated LA shape as a marker of AtCM and occult AF-related substrate [88,89]. These findings broaden LASP from a recurrence marker to a potential imaging phenotype of atrial disease.

Nevertheless, the evidence is not uniform. Bossard et al. found that conventional and 3D echocardiographic parameters predicted recurrence after AF ablation, but the incremental value of 3D geometry was not consistently superior [82]. Den Uijl et al. showed that LAV, sphericity, and fibrosis were interrelated, with LAV emerging as the strongest predictor in their cohort [81]. Guo et al. found that LAV, sphericity, and reduced LA function were associated with late recurrence, but not all geometric indices retained independent prognostic value [90]. Mulder et al. reported no significant difference in LA sphericity between AF patients and controls despite clear differences in LA diameter and LAV [91]. These discrepancies emphasize that LASP is promising but method-sensitive, and its clinical interpretation depends on imaging modality, segmentation boundaries, rhythm at acquisition, AF phenotype, endpoint definition, and calculation formula.

### 4.3. ASI: Asymmetric Anterior Remodeling

ASI captures directional LA remodeling by quantifying the proportion of total LA volume located anterior to a standardized anatomical plane. In its commonly used CT-derived formulation, the LA is divided into anterior and posterior compartments by a plane parallel to the posterior wall and passing between the PV ostia and the LAA; ASI is then calculated as anterior LA volume divided by total LA volume [13,30,92,93]. This metric reflects the observation that LA enlargement in AF is not simply spherical expansion but often asymmetric deformation shaped by surrounding anatomical constraints, including the spine, descending aorta, sternum, PVs, LAA, and mediastinal structures.

Nedios et al. provided foundational evidence that LAV and 3D LA structure differ across AF phenotypes and that asymmetric remodeling has prognostic value after ablation [30]. In subsequent work, asymmetrical LA remodeling was associated with diastolic dysfunction and long-term ablation outcomes, supporting the concept that anterior LA expansion may represent a geometric signature of chronic filling-pressure burden and progressive AtCM [13]. The mechanistic link between anatomy and function was strengthened by studies showing that altered LA activation patterns, particularly U-shaped activation, are associated with increased asymmetry and diastolic dysfunction [92]. In patients with AF and hypertrophic cardiomyopathy (HCM), LA remodeling was also characterized by asymmetric anterior dilatation, suggesting that ASI may be particularly informative in disease-specific substrates marked by impaired LV compliance and elevated filling pressures [93].

The ASI evidence base remains smaller than that for LAV or LASP. Guo et al. did not identify ASI as an independent predictor of late recurrence after catheter ablation, highlighting potential context dependency [90]. This discrepancy may reflect differences in AF subtype composition, imaging protocol, segmentation method, chamber size, diastolic function, and ablation strategy. ASI should therefore be interpreted as a regional remodeling marker that may complement, rather than replace, LAV and LASP.

### 4.4. AEI: Eccentric Elongation and Anisotropic Deformation

AEI is designed to quantify eccentric chamber elongation and is generally conceptualized as the ratio between the maximum LA diameter and the mean LA diameter derived from 3D reconstruction. Whereas LASP captures global spherical transformation and ASI captures preferential anterior remodeling, AEI captures anisotropic stretching along a dominant axis. A higher AEI may indicate an elongated or ovoid chamber phenotype, while a lower eccentricity profile may indicate more spherical expansion.

Compared with LAV, LASP, and ASI, AEI has been less extensively validated in AF-specific ablation cohorts. Eccentricity-based LA analysis has been studied in other hemodynamic settings, such as chronic mitral regurgitation, where progressive volume overload is associated with altered LA shape and impaired function [94]. In AF, AEI should therefore be regarded as an emerging descriptor rather than an established prognostic biomarker. Its potential value lies in distinguishing different geometric phenotypes among patients with similar LAV: some atria remodel toward spherical enlargement, whereas others develop eccentric elongation. These phenotypes may have different implications for wall stress distribution, posterior wall configuration, mitral isthmus geometry, catheter orientation, and regional conduction.

### 4.5. PV Anatomy, Ostial Geometry, and Orientation

PV anatomy is central to AF because PV myocardial sleeves are key triggers, and PVI remains the cornerstone of catheter ablation. Three-dimensional CT and CMR enable quantitative assessment of PV number, common trunks, accessory veins, ostial area, ovality, angulation, takeoff direction, antral dimensions, and spatial orientation relative to the LA body [129]. These features are clinically meaningful because they influence transseptal access, catheter stability, balloon occlusion, lesion contiguity, and the likelihood of PV reconnection [46,99].

Several primary studies support the procedural relevance of PV geometry. Sorgente et al. showed that PV ostial shape and orientation predicted the degree of cryoballoon occlusion, with PV orientation playing an important role in procedural performance [37]. Sohns et al. reported that PV anatomy assessed by MDCT predicted freedom from atrial tachyarrhythmia after remote magnetic navigation-guided circumferential PVA [95]. Wei et al. found that superior PV enlargement, together with LAD, AF duration, and AF type, independently predicted recurrence after RF ablation [96]. Knecht et al. further linked LA anatomy, AF burden, and P-wave duration with single-procedure success after PVI, suggesting that structural and electrical phenotypes converge in determining procedural response [97]. CT-based cryoballoon studies have also shown that anatomical difficulty, ostial dimensions, PV variants, and RSPV diameter may affect acute isolation and long-term outcomes [98,99,100]. More recently, radiomic analyses of PV morphology have shown that CT-derived PV shape and texture features may identify patients at increased risk of recurrence after ablation [101].

### 4.6. LAA Morphology and Ostial Geometry

The LAA is the dominant site of thrombus formation in AF and has substantial interindividual anatomical variability [130,131]. Three-dimensional imaging can quantify LAA morphology, number of lobes, bend angle, ostial dimensions, takeoff height, landing-zone geometry, depth, volume, and spatial relationship to the left superior PV and left lateral ridge. These features are relevant to thromboembolic risk, LAA closure (LAAC) planning, and possibly post-ablation recurrence.

Di Biase et al. reported that chicken-wing LAA morphology was associated with lower embolic risk than non–chicken-wing morphologies after adjustment for clinical risk factors [102]. Nedios et al. subsequently showed that thromboembolic events after AF ablation were associated with AF recurrence and higher LAA takeoff, whereas categorical LAA morphology alone was not independently predictive [103]. Smit et al. found that LA and LAA anatomical features were associated with previous stroke/TIA in AF cohorts, reinforcing the concept that appendage geometry should be interpreted as part of the broader LA phenotype [104]. Statistical shape analysis has further suggested that quantitative LAA shape modeling may predict stroke in AF more robustly than categorical morphology labels [105].

The LAA may also relate to ablation outcomes [132]. Gong et al. reported an association between LAA morphology and AF recurrence after RF ablation [106]. Simon et al. showed that LAA enlargement was associated with recurrence in persistent AF [36]. Papathanasiou et al. found that LAA morphofunctional indices were associated with late arrhythmia recurrence after AF catheter ablation [107]. Additional CT-based work has suggested that anatomical proximity or abutment between the LAA and left superior PV may predict recurrence after point-by-point PVI [108], while recent data have implicated windsock-type LAA morphology as a potential recurrence marker [109]. Taken together, these findings suggest that LAA assessment should move beyond simple morphology labels toward quantitative ostial, volumetric, spatial, and functional characterization.

### 4.7. Posterior Wall, LA Roof, and Left Lateral Ridge Geometry

Regional LA geometry is particularly relevant around the posterior wall, roof, mitral isthmus, and left lateral ridge [38,39,133,134]. The posterior wall is contiguous with the PV antra, closely related to the esophagus, and frequently implicated in persistent AF substrate. Three-dimensional reconstruction can quantify posterior wall size, curvature, flattening, relationship to PV ostia, and regional deformation. Similarly, the LA roof and left lateral ridge may influence conduction, catheter stability, ablation line design, and residual arrhythmogenic substrate.

Kurotobi et al. evaluated the shape of the LA roof as a novel index reflecting electrophysiological and structural characteristics of the PV–LA complex, showing that regional shape analysis can provide substrate-relevant information beyond global chamber size [110]. Shape-statistical analysis by Jia et al. found that LA regions associated with ablation failure clustered around the postero-inferior LA, mitral isthmus, and left inferior PV; importantly, the CT-derived shape score predicted ablation failure independently of AF persistence and LAVI [14]. Nedios et al. also demonstrated that low-voltage areas were associated with regional wall deformation and LA shape, providing direct evidence that anatomical remodeling and electrical substrate are linked [111]. More recent CT work has identified left lateral ridge geometry as an independent predictor of recurrence after RF ablation, further supporting the procedural relevance of regional LA anatomy [112].

These studies indicate that regional geometry may be especially important when global metrics are equivocal. Patients with similar LAV and LASP may differ substantially in posterior wall configuration, roof shape, and ridge anatomy, with potential consequences for lesion delivery and arrhythmia recurrence.

### 4.8. LA Wall Thickness and Lesion-Relevant Anatomy

LA wall thickness is an increasingly important geometric metric because lesion formation, transmurality, and reconnection risk depend not only on chamber shape but also on local tissue thickness. CT studies have shown marked regional variability in LA wall thickness, particularly around the PV antra, ridge, septum, roof, and posterior wall [113]. Beinart et al. demonstrated substantial inter- and intra-patient variability in LA wall thickness before PVI, establishing the anatomical premise for thickness-aware ablation planning [113]. Inoue et al. found that increased LA wall thickness was associated with recurrence and PV reconnection after RF ablation, suggesting that thicker atrial regions may require different lesion parameters [114].

Several subsequent studies have explored this concept in greater detail. Mulder et al. evaluated local LA wall thickness and acute PV reconnection after ablation-index-guided PVI, showing that wall thickness may affect lesion durability even when contemporary lesion-quality indices are used [115]. Teres et al. specifically examined LA wall thickness at the PV component and its relationship with PV reconnection [116]. Oh et al. used 3D wall-thickness mapping to relate LA wall thickness to reconnection after PVI [117]. Lee et al. reported that tailored ablation-index targets based on CT-derived LA wall thickness were feasible and reduced acute PV reconnection, supporting the translational potential of geometry-informed lesion delivery [118]. Conversely, Boussoussou et al. found that LA wall thickness did not influence acute procedural success under a standardized ablation-index protocol, although PV dimensions, particularly RSPV diameter, affected first-pass isolation [100].

Emerging CT/CMR studies have extended wall-thickness analysis to broader electroanatomic remodeling. Recent data suggest that LA wall thickness may correlate with AF progression, low-voltage substrate, and regional electroanatomic remodeling, although acquisition, segmentation, and thresholding remain challenging [119,120]. Overall, LA wall thickness represents a bridge between anatomy and ablation biophysics and may become particularly relevant for personalizing lesion delivery across catheter ablation platforms.

### 4.9. Surface Curvature, Fractal Features, Radiomics, and AI-Derived Shape Descriptors

Advanced computational approaches extend 3D geometry beyond prespecified metrics. Surface curvature can capture regional ridges, flattening, concavity, and deformation not reflected by LAV, LASP, ASI, or AEI. Statistical shape modeling and atlas-based approaches can identify spatial deformation patterns associated with recurrence, often without requiring manual selection of a single anatomical index. Varela et al. used computational anatomy of preprocedural LA geometry and showed that shape analysis improved prediction of AF recurrence after ablation [121]. Bieging et al. used particle-based modeling to demonstrate that LA shape predicted recurrence and added prognostic value to fibrosis assessment [42]. Jia et al. developed a CT-derived LA shape score that predicted ablation failure independently of LAVI and AF persistence [14].

Radiomic and fractal features provide another layer of quantification by measuring shape complexity and image texture. Firouznia et al. demonstrated that machine learning-derived fractal features of LA and PV shape and texture from CT scans were associated with recurrence after AF ablation [122]. Atta-Fosu et al. used machine learning and CT atlas features to identify recurrence-associated shape differences near the LAA and PVs [123]. Roney et al. combined population data with virtual cohorts of patient-specific LA models to predict recurrence, illustrating how anatomical modeling can be integrated with mechanistic simulation [124]. Deep-learning studies using 3D reconstructed LA images have also shown feasibility for predicting recurrence after PVI, while multimodal machine-learning frameworks incorporating CT-derived variables have reported promising performance [125,126].

## 5. Three-Dimensional Geometry as a Marker of AtCM

Three-dimensional LA geometry is best interpreted not as a static anatomical descriptor, but as the visible end-product of AtCM. The atrium remodels in response to a convergent set of insults, including pressure and volume overload, aging, hypertension, obesity, diabetes, sleep-disordered breathing, valvular disease, heart failure, inflammation, epicardial adiposity, and sustained arrhythmia exposure. These processes act at the tissue level through myocyte hypertrophy, interstitial fibrosis, extracellular matrix expansion, adipose infiltration, microvascular dysfunction, autonomic remodeling, and impaired atrial compliance [40,135]. Three-dimensional deformation therefore provides a macroscopic phenotype of microscopic disease: the shape of the LA may encode cumulative information about wall stress, regional stretch, mechanical failure, conduction heterogeneity, fibrotic replacement, and the probability of rhythm-control failure.

### 5.1. LA Shape and Wall Stress

The mechanistic relevance of LA shape derives from the relationship between chamber geometry and mechanical load. Wall stress is not determined by LA volume alone, but by the interaction among local curvature, chamber pressure, regional wall thickness, and anatomical constraints. Patient-specific modeling studies have shown that wall stress is heterogeneously distributed across the LA, with high-stress regions frequently located around the PV antra, posterior wall, septum, anterior wall, and appendage ridge [136]. Importantly, areas of increased mechanical stress have been linked to lower local voltage, suggesting that chronic stretch may promote regional electrophysiological remodeling [3]. Subsequent work has associated estimated LA wall stress with rhythm outcomes after catheter ablation, supporting the concept that mechanical loading is not merely a consequence of AF but may participate in substrate progression [137].

This framework explains why global enlargement is an incomplete marker of atrial disease. Two atria with the same volume may have substantially different stress distributions depending on curvature, regional deformation, and wall thickness. Progressive spherical or eccentric deformation may therefore be interpreted as a geometric adaptation to chronic load, while localized deformation may indicate spatially concentrated stress [138]. In this sense, LA shape operates as a biomechanical biomarker: it reflects how atrial tissue has accommodated cumulative hemodynamic burden and where structural vulnerability may have developed.

### 5.2. LA Asymmetry and Thoracic Constraints

Atrial remodeling is anatomically constrained. The LA cannot enlarge freely in all directions because it is bounded by the spine, descending aorta, pulmonary veins, pericardium, sternum, and adjacent mediastinal structures. Consequently, AF-related enlargement often follows nonuniform patterns rather than simple spherical dilatation. Early echocardiographic observations described trapezoidal remodeling, indicating that atrial expansion may preferentially involve certain regions rather than the entire chamber uniformly [29]. CT-based studies later confirmed that atrial remodeling differs across AF phenotypes and may involve preferential anterior deformation, particularly in more advanced disease [13,30].

This directional deformation is biologically relevant because it reflects the interaction between intracardiac loading and extracardiac restraint. Chronic elevation in LV filling pressure may drive LA expansion, but the final geometric phenotype is shaped by where the atrium is allowed to expand. Studies linking asymmetric remodeling with diastolic dysfunction, altered activation patterns, and long-term ablation outcomes suggest that asymmetry is not simply an anatomical curiosity but a marker of advanced atrial remodeling [92]. In disease-specific settings such as HCM, preferential anterior remodeling further supports the view that geometric deformation reflects the mechanical consequences of impaired ventricular compliance and atrial pressure loading [93].

### 5.3. Geometry–Function Coupling: Strain, Reservoir Function, and Activation

AtCM is not defined by structure alone. Loss of LA reservoir, conduit, and contractile function may precede, accompany, or outpace overt geometric enlargement. Speckle-tracking and CMR feature-tracking studies have consistently shown that reduced LA strain and impaired reservoir function predict AF recurrence after catheter ablation [139,140,141,142]. These findings are mechanistically important because they indicate that the remodeled atrium is not only larger or differently shaped, but also mechanically dysfunctional.

Geometry and function are therefore interdependent. A more deformed atrium may reflect increased stiffness, impaired relaxation, reduced compliance, and abnormal mechanical activation [140,143,144]. Conversely, impaired reservoir function may accelerate chamber deformation by increasing local stretch and promoting further remodeling. Studies of intra-atrial dyssynchrony have shown that abnormal mechanical timing during sinus rhythm predicts recurrence after ablation, suggesting that atrial activation and contraction abnormalities can persist even when surface rhythm appears stable [140]. Direct evidence linking LA sphericity with impaired strain and strain-rate parameters further supports the concept that geometric remodeling and mechanical failure are coupled manifestations of the same disease process [87].

This geometry–function coupling has practical implications. A patient with modest LA enlargement but severely impaired strain may have more advanced AtCM than volume alone suggests. Conversely, geometric remodeling interpreted together with strain, emptying fraction, or activation delay may provide a more complete substrate phenotype than either structure or function alone.

### 5.4. Geometry–Fibrosis Coupling: LGE, Low Voltage, and Electroanatomic Substrate

Fibrosis provides the histological substrate that links geometric remodeling with electrical instability. LGE-CMR studies established that LA fibrosis can be detected noninvasively and that greater fibrotic burden is associated with higher recurrence after catheter ablation [47,145,146]. The DECAAF study demonstrated a graded relationship between atrial fibrosis and post-ablation arrhythmia recurrence, supporting fibrosis as a clinically relevant substrate marker [47]. However, DECAAF II also showed that MRI-guided fibrosis ablation did not significantly improve recurrence outcomes compared with conventional ablation, indicating that substrate identification and effective substrate modification are not equivalent [48].

The relationship between geometry and fibrosis is likely bidirectional. Chronic stretch and wall stress may stimulate profibrotic signaling, while fibrosis increases atrial stiffness and promotes further deformation. Imaging studies have shown that LAV, LA shape, and fibrosis are interrelated, suggesting that geometric remodeling and tissue remodeling are overlapping expressions of AtCM rather than independent phenomena [81]. At the electrical level, low-voltage areas provide an invasive correlate of diseased substrate and have been associated with recurrence after catheter ablation [53,54,55,66]. The correlation between LGE-CMR and voltage mapping is imperfect because each modality measures a different biological signal and is influenced by technical factors such as spatial resolution, rhythm, electrode configuration, wall thickness, and registration accuracy [147]. Nevertheless, studies linking regional deformation, low-voltage substrate, and LA shape support a spatial relationship between anatomical remodeling and electrical disease [111].

Additional substrate layers reinforce this concept. Regional wall-thickness variation may influence voltage amplitude, lesion formation, and reconnection risk, while epicardial adipose tissue has been associated with LA dysfunction, low-voltage zones, and recurrent AF after ablation [148,149,150,151]. Thus, 3D geometry should be considered the anatomical scaffold on which fibrosis, voltage abnormalities, adipose infiltration, wall-thickness heterogeneity, and mechanical dysfunction are superimposed.

### 5.5. Geometry and AtCM Staging

The AtCM framework provides the most coherent context for interpreting 3D LA geometry. Consensus definitions describe AtCM as a complex of structural, architectural, contractile, or electrophysiological changes with potential clinical relevance [40,135]. Within this framework, geometry may serve as the structural axis of disease staging. Early AtCM may be characterized by subtle mechanical impairment, regional deformation, dyssynchrony, or abnormal PV–LA relationships before overt chamber enlargement. Intermediate disease may manifest as increasing LAV, progressive shape deformation, anterior or regional asymmetry, and early low-voltage or fibrotic substrate. Advanced disease may involve extensive fibrosis, diffuse low-voltage areas, severe reservoir dysfunction, marked stiffness, wall-thickness heterogeneity, and limited reverse remodeling after rhythm-control therapy.

Importantly, these stages are unlikely to be linear or uniform. Different upstream drivers may produce different geometric phenotypes. Hypertension and diastolic dysfunction may promote pressure-related asymmetric remodeling; obesity may amplify epicardial adipose-driven inflammatory remodeling; valvular disease may favor volume-related chamber deformation; and persistent AF may accelerate fibrosis, electrical remodeling, and mechanical failure [152,153,154,155]. Therefore, 3D geometry should not be interpreted as a single marker of disease severity, but as a phenotype that reflects the dominant remodeling pathway in an individual patient.

This mechanistic interpretation is particularly relevant in the PFA era. PFA may improve safety and lesion selectivity [18], but it does not eliminate the underlying AtCM that predisposes to recurrence. Patients with advanced geometric deformation may remain vulnerable to recurrent AF because their substrate extends beyond PV triggers and reflects diffuse atrial disease. Prospective studies are therefore needed to determine whether CT-derived 3D geometry can identify the AtCM phenotype most likely to recur after PFA and whether geometry can guide patient selection, monitoring intensity, or adjunctive substrate assessment.

## 6. Three-Dimensional Geometry and Catheter Ablation Outcomes

Catheter ablation outcomes are determined not only by lesion delivery, energy source, and procedural endpoints, but also by the pre-existing atrial substrate. Three-dimensional LA geometry is therefore clinically relevant because it may identify patients in whom AF is still predominantly trigger-driven and PV-dependent, as opposed to patients with advanced AtCM, diffuse remodeling, posterior wall disease, or non-PV substrate. In this context, 3D geometry should be viewed as an imaging-derived phenotype of ablation vulnerability: it may predict recurrence, inform the need for more intensive monitoring, and help determine whether anatomical PVI alone is likely to be sufficient.

Key studies linking advanced atrial geometric, functional, electrophysiological, and computational phenotyping with rhythm-control outcomes after AF ablation are summarized in Table 3.

### 6.1. Pre-Ablation Geometric Phenotype and Recurrence Risk

Pre-ablation LA size remains one of the most reproducible predictors of AF recurrence after catheter ablation. LAV and LAVI outperform linear LA diameter because they better capture global chamber burden, and multiple primary studies and meta-analyses have shown that larger LA volume is associated with lower arrhythmia-free survival after RF ablation and PVI [24,51,80,81]. However, the limitations of volume-only assessment are clinically important. Two patients with comparable LAVI may have different recurrence risk if one has preserved atrial architecture whereas the other has spherical remodeling, anterior asymmetry, posterior wall deformation, unfavorable PV anatomy, or LAA enlargement.

Shape-based studies have therefore refined the prognostic interpretation of LA enlargement. Bisbal et al. introduced LA sphericity as a marker of atrial remodeling and showed that greater spherical remodeling predicted AF recurrence after ablation [31]. The multicenter LAGO-AF study confirmed the prognostic relevance of LA geometry, identifying sphericity and AF phenotype as major predictors of outcome [33]. CMR-based work by Nakamori et al. demonstrated that geometric remodeling added incremental value for predicting late recurrence after PVI, while particle-based and statistical shape-modeling studies by Bieging et al., Varela et al., and Jia et al. showed that global and regional LA shape can predict recurrence beyond conventional clinical variables and LAVI [14,32,121]. Importantly, Jia et al. identified recurrence-associated shape patterns around the postero-inferior LA, mitral isthmus, and left inferior PV, suggesting that risk is not only related to global chamber enlargement but also to regional deformation [14].

Asymmetric remodeling provides another prognostic layer. Nedios et al. showed that LA volume and 3D structure differ across AF phenotypes and that asymmetrical remodeling may predict ablation outcomes [30]. Subsequent studies linked anterior asymmetric dilation with diastolic dysfunction, altered LA activation, and adverse long-term rhythm outcomes [13,92]. These findings are particularly relevant for patients with persistent AF or comorbidity-driven atrial disease, in whom recurrence may reflect a substrate that extends beyond the PVs.

PV anatomy and LAA geometry further modify procedural risk. CT studies have shown that PV ostial size, orientation, ovality, common trunks, accessory veins, and antral geometry may influence cryoballoon occlusion, catheter stability, lesion delivery, and recurrence [37,96,97,99]. LAA parameters also appear relevant: although simple categorical morphology has yielded inconsistent results, LAA volume, orifice area, takeoff height, and spatial relationship to the left superior PV may be associated with recurrence and thromboembolic risk [102,103,104,106,107]. Posterior wall, roof, and lateral ridge anatomy are similarly important because these regions influence lesion-set design, catheter contact, conduction channels, and residual substrate [110,112]. Thus, the most useful pre-ablation geometric phenotype is not a single variable but an integrated assessment of chamber burden, shape deformation, venous anatomy, appendage geometry, and regional substrate-relevant anatomy.

### 6.2. Geometry and Ablation Strategy Selection

The main clinical question is whether geometry can move from risk prediction to strategy selection. Current randomized evidence cautions against empirical substrate ablation in all patients. STAR AF II showed no benefit from adding linear ablation or complex fractionated atrial electrograms (CFAE) ablation to PVI in persistent AF [166]. CAPLA similarly showed that empirical posterior wall isolation added to PVI did not improve 12-month freedom from atrial arrhythmia in first-time persistent AF ablation [167]. These trials support an important principle: more ablation is not necessarily better when adjunctive lesion sets are applied anatomically rather than according to patient-specific substrate.

Three-dimensional geometry may help define which patients should remain in a PVI-only pathway and which patients warrant more intensive substrate assessment. A small, minimally deformed LA with favorable PV anatomy and preserved function may represent a predominantly PV-trigger phenotype, for which durable PVI alone may be sufficient. Conversely, marked LA enlargement, high sphericity, anterior asymmetry, posterior wall deformation, extensive LAA enlargement, unfavorable PV anatomy, or regional wall-thickness heterogeneity may suggest advanced AtCM and a greater probability of non-PV substrate. In such patients, geometry should not automatically mandate empiric posterior wall isolation or linear lesions; rather, it should prompt a more deliberate substrate evaluation using EAM, voltage mapping, CMR fibrosis assessment, or intensified post-ablation rhythm surveillance.

Voltage-guided ablation provides a model for this individualized approach. Non-randomized and randomized studies have suggested that LVA-guided substrate modification can reduce recurrence in selected patients with persistent AF and demonstrable low-voltage substrate [53,168]. The ERASE-AF trial reported improved outcomes when PVI was combined with individualized ablation of low-voltage myocardium in persistent AF [168]. However, more recent randomized data from SUPPRESS-AF showed that, among persistent AF patients with LVAs after PVI, additional LVA ablation did not uniformly improve outcomes across all patients, emphasizing that substrate-guided approaches are highly dependent on patient selection, substrate extent, mapping quality, and lesion strategy [169]. Geometry may therefore be most valuable as a triage tool: it can identify patients in whom PVI-only ablation is likely to be adequate, and those in whom further substrate interrogation is justified.

### 6.3. Geometry and Energy Modality: Thermal Ablation Versus PFA

Most geometric predictors of ablation outcome were derived from RF or cryoballoon cohorts, and their direct extrapolation to PFA should therefore be cautious. Recent comparative evidence from randomized trials indicates that ablation energy modalities differ in procedural performance, AF burden reduction, and safety outcomes, reinforcing the need to interpret geometry-derived predictors within an energy-specific framework [170]. Major PFA studies, including PULSED AF, ADVENT, inspIRE, MANIFEST-PF, and MANIFEST-17K, have established the clinical effectiveness and favorable safety profile of PFA, but they were not designed to determine how CT-derived LA geometry modifies lesion durability, recurrence risk, or post-ablation remodeling [18,171,172,173,174]. In thermal ablation, LA geometry influences outcome mainly by modifying energy delivery. During RF ablation, regional wall thickness, ridge anatomy, posterior wall configuration, catheter orientation, contact force, stability, interlesion distance, and convective cooling may affect lesion depth and durability [113,118,175,176,177]. During cryoballoon ablation, PV ostial size, ovality, angulation, common trunks, and antral orientation influence balloon occlusion, circumferential cooling, and the risk of incomplete isolation [37,99,178,179]. Thus, in thermal technologies, adverse geometry may predict recurrence partly because it creates anatomical conditions that favor non-transmural lesions, conduction gaps, or PV reconnection.

In PFA, geometry remains important, but through different mechanisms. PFA produces non-thermal electroporation, in which lesion formation depends on electric-field distribution, electrode configuration, electrode–tissue proximity, catheter orientation, pulse waveform, pulse burden, tissue conductivity, and blood-pool exposure rather than conductive heating or freezing [180,181,182]. Therefore, LA enlargement, spherical remodeling, wide PV antra, broad posterior wall geometry, prominent left lateral ridge anatomy, or highly angulated PV takeoff may influence the spatial relationship between the PFA catheter, atrial tissue, and circulating blood pool. This may modify field penetration, lesion overlap, current shunting, and the balance between effective myocardial electroporation and non-target energy dissipation. PFA may reduce some limitations of thermal energy, but it should not be considered geometry-independent.

Importantly, the influence of LA geometry may not be uniform across PFA platforms. Single-shot systems, including pentaspline and balloon-in-basket designs, are intended to achieve circumferential PVI through anatomically conforming multielectrode configurations, whereas focal lattice-tip systems permit anatomy-tailored lesion deployment but require sequential catheter manipulation and contiguous lesion placement [183,184]. In a recent multicentre comparison of 447 patients with paroxysmal AF, lattice-tip and pentaspline PFA achieved similarly high procedural and 1-year clinical success, whereas the lattice-tip workflow was associated with longer procedure and LA dwell times but substantially shorter fluoroscopy exposure, illustrating that catheter architecture may modify procedural efficiency even when clinical effectiveness is comparable [185]. Consistent with this platform-specific interaction, a recent CT-based analysis found only a limited association between PV geometric characteristics and reconnection after pentaspline PFA, suggesting relative adaptability of this configuration to anatomical variation [186]. PFA may also attenuate, but does not eliminate, the influence of wall thickness: lesion depth remains dependent on electric-field dose and electrode–tissue proximity, while recent fixed-loop data suggest reliable transmural lesion formation in atrial walls <4 mm thick but persistent limitations in thicker tissue [187,188]. Thus, LA geometry in the PFA era should be regarded as a technology-specific determinant of device deployment, procedural efficiency, lesion coverage, and potentially durability, rather than as a universal predictor across PFA platforms.

These technology-specific effects also have implications for how geometry should be interpreted prognostically. In RF or cryoballoon cohorts, recurrence in patients with adverse LA geometry may reflect both technical lesion limitations and advanced AtCM. With PFA, if durable PVI is achieved more consistently, recurrence associated with high LAV/LAVI, increased LASP, anterior asymmetry, posterior wall deformation, or LAA enlargement may more specifically indicate diffuse substrate, non-PV triggers, or a less PV-dependent AF phenotype. This distinction is clinically important because geometry may help separate patients in whom recurrence is driven by lesion failure from those in whom recurrence reflects advanced atrial disease.

PFA may also modify the interpretation of post-ablation remodeling. After thermal ablation, reductions in LA volume may reflect true rhythm-related reverse remodeling, but also scar contraction, atrial debulking, or impaired compliance after extensive ablation [189,190]. In contrast, experimental and emerging comparative clinical data suggest that PFA may preserve atrial mechanical architecture more effectively than thermal ablation, with less chronic fibrotic remodeling and less LA volume reduction after extensive ablation beyond the PVs [191,192,193,194]. Future studies should therefore evaluate whether CT-derived three-dimensional LA metrics predict PFA-specific endpoints, including first-pass isolation, lesion durability, AF burden, recurrence phenotype, LA strain, and the need for repeat ablation.

### 6.4. Geometry and Reverse Remodeling After Ablation

Successful rhythm control can induce LA reverse remodeling, but the interpretation of post-ablation geometric change is complex. Early studies showed that restoration and maintenance of sinus rhythm after catheter ablation can reduce LA size and improve mechanical function [195,196,197]. LA strain and reservoir function have been shown to predict the likelihood of reverse remodeling, suggesting that pre-existing mechanical reserve determines whether the atrium can recover after rhythm control [141]. Serial imaging studies further support that reductions in LAV and improvements in strain are associated with better rhythm outcomes [198,199].

However, not all LA volume reduction represents true biological recovery. Thermal ablation creates scar, and extensive lesion sets may reduce chamber compliance or induce stiff LA physiology [200]. In this setting, a smaller LA after ablation may reflect a mixture of beneficial reverse remodeling, scar-mediated contraction, atrial debulking, and impaired compliance. Studies of post-ablation LA stiffness and stiff LA physiology have shown that extensive LA ablation can increase LA pressure and impair atrial reservoir mechanics, particularly when substrate modification extends beyond PVI [189,190]. This distinction is critical when interpreting geometry: favorable remodeling should ideally combine reduced chamber size with improved strain, preserved compliance, lower AF burden, and better symptoms; isolated volume reduction without functional recovery may instead reflect iatrogenic scarring.

PFA may help separate these mechanisms. Experimental work showed that PFA can prevent chronic atrial fibrotic changes and restrictive mechanics after ablation, and clinical studies suggest that PFA-based posterior wall isolation may preserve LA physiology better than thermal strategies [191,194]. Recent PFA studies evaluating LA volume, strain, fibrosis, and function after PVI or ablation beyond PVs are therefore especially relevant because they may clarify whether post-PFA geometric remodeling reflects true atrial recovery rather than scar contraction [192,193]. For future studies, reverse remodeling should be defined multidimensionally, incorporating LAV/LAVI, LA strain, symptom burden, AF burden, and, where available, fibrosis or voltage data.

### 6.5. Geometry, Early Recurrence, and Long-Term Rhythm Outcomes

The blanking period has traditionally been interpreted as a phase during which inflammation, transient autonomic changes, edema, and lesion maturation may produce arrhythmias that do not necessarily indicate ablation failure [17]. However, accumulating evidence indicates that early recurrence is not benign, especially when episodes occur later within the blanking period or with increasing burden [201,202,203,204]. Continuous monitoring studies have shown that recurrence timing and AF burden during blanking provide more prognostic information than a simple binary recurrence definition [203,204]. This is particularly relevant because intermittent ECG or Holter monitoring may underestimate early arrhythmic activity.

PFA has renewed interest in the blanking-period concept. Because PFA produces different lesion biology and less thermal inflammation, early recurrence after PFA may have a different meaning than early recurrence after RF or cryoballoon ablation. The admIRE subanalysis showed that early recurrence after PFA was strongly associated with late recurrence, and patients with early recurrence had substantially lower freedom from documented recurrence at 12 months than those without early recurrence [205]. Contemporary discussions therefore question whether the conventional 90-day blanking period should be applied identically across energy modalities [206,207,208].

Three-dimensional geometry may help interpret early recurrence after ablation. In patients with favorable geometry and limited atrial remodeling, early episodes may be more likely to represent transient post-procedural phenomena [24,201]. In contrast, early recurrence in a patient with high LAV/LAVI, spherical remodeling, anterior asymmetry, posterior wall deformation, LAA enlargement, or extensive substrate markers may indicate persistent AtCM and a higher probability of late failure. This creates a strong rationale for combining pre-ablation geometry with early post-ablation rhythm monitoring. A geometry-informed monitoring strategy could classify patients into low-risk, intermediate-risk, and high-risk trajectories based on baseline CT phenotype and blanking-period AF burden.

This approach is particularly aligned with wearable and smartwatch-based follow-up. Continuous or near-continuous rhythm monitoring during the blanking period can quantify episode timing, frequency, duration, and burden rather than relying only on symptomatic recurrence or scheduled ECGs. When integrated with CT-derived 3D geometry, early rhythm data may distinguish transient post-ablation instability from substrate-driven recurrence. In the PFA era, this combined framework is clinically attractive: CT geometry defines the pre-ablation AtCM phenotype, while early wearable-detected arrhythmia burden tests whether that substrate remains electrically active after lesion delivery. Prospective studies are needed to determine whether this combined anatomical–rhythm phenotype can predict late recurrence, guide follow-up intensity, or identify patients who may benefit from early reintervention or adjunctive substrate evaluation.

## 7. Limitations and Future Directions

The clinical translation of 3D LA geometry in AF remains constrained by methodological heterogeneity, limited external validation, and uncertainty regarding clinical actionability. A first limitation concerns image acquisition and reconstruction. Geometric indices are sensitive to rhythm during imaging, cardiac phase, spatial resolution, contrast timing, motion correction, segmentation thresholds, smoothing algorithms, and whether the PVs and LAA are included in the atrial shell [209,210,211,212]. These technical choices can alter LAV/LAVI, LASP, ASI, AEI, wall thickness, and regional surface descriptors, limiting comparability across studies. Future work should therefore prioritize standardized CT and CMR acquisition protocols, harmonized segmentation rules [213], reproducibility benchmarks, and explicit reporting of all preprocessing decisions [119,211,212].

A second limitation is conceptual. Three-dimensional geometry provides a structural phenotype of atrial remodeling, but it does not directly measure fibrosis, inflammation, adiposity, conduction velocity, autonomic tone, or mechanical reserve. Accordingly, geometry should not be interpreted as a standalone definition of AtCM. Future phenotyping should integrate CT-derived anatomy with CMR tissue characterization, EAM substrate information, echocardiographic strain, circulating biomarkers, clinical risk factors, DAT, and longitudinal rhythm monitoring, given that longer AF history before ablation has been associated with higher arrhythmia recurrence and worse clinical outcomes after catheter ablation [40,214]. Such multimodal models may be particularly relevant after ablation, where the distinction between transient post-procedural arrhythmia, persistent substrate activity, and durable rhythm control cannot be inferred from baseline anatomy alone [215].

A third limitation is translational. Most available geometry-based studies are observational, retrospective, and single-center. As a result, current geometric markers should be regarded primarily as risk phenotypes rather than treatment-selection tools. Demonstrating that a specific geometric pattern predicts recurrence does not prove that a geometry-guided strategy improves outcomes. Future studies should therefore test whether 3D geometry can prospectively alter management: selecting patients for PVI-only versus intensified substrate assessment, tailoring follow-up intensity, refining post-ablation rhythm surveillance, or identifying patients who may benefit from earlier repeat evaluation. This is especially important in the PFA era, because predictors derived from thermal ablation cohorts may not translate directly to a non-thermal energy source with distinct lesion biology and tissue selectivity.

Artificial intelligence (AI) and machine learning (ML) offer a pathway to scalable geometric phenotyping, but they also introduce new risks [216]. Automated segmentation may reduce observer dependency and enable large-scale extraction of LA, PV, LAA, and wall-thickness metrics, while radiomics, statistical shape modeling, deep learning, and digital twin approaches may identify latent anatomical phenotypes that are not captured by predefined indices [217,218,219,220,221]. However, many AI models remain vulnerable to small sample size, data leakage, class imbalance, scanner dependence, segmentation variability, poor calibration, and limited interpretability. Future studies should therefore include external validation, calibration analysis, decision-curve analysis, subgroup performance assessment, model updating plans, and transparent reporting of preprocessing, feature selection, and missing-data handling [222,223].

For implementation, geometry-based and AI-enabled models should be developed according to established reporting and evaluation frameworks. Prediction models should follow TRIPOD+AI, risk-of-bias assessment should use PROBAST+AI, medical-imaging AI studies should follow CLAIM, early clinical evaluation should follow DECIDE-AI, and interventional trials of AI-supported workflows should follow CONSORT-AI and SPIRIT-AI [224,225,226]. Broader trustworthy-AI principles, including fairness, universality, traceability, usability, robustness, and explainability, should also be incorporated before deployment [16]. These standards are essential because a model trained in one imaging environment may perform poorly when applied to different scanners, segmentation pipelines, populations, rhythm-monitoring strategies, or ablation platforms.

Future research should therefore move from isolated geometric indices toward validated, interpretable, and clinically actionable atrial phenotypes. Key priorities include: standardized LA/PV/LAA segmentation; harmonized definitions of LASP, ASI, AEI, and wall thickness; open benchmarking datasets; multicenter prospective validation; integration with function, fibrosis, voltage, biomarkers, DAT, and wearable-derived AF burden; and dedicated PFA-era studies evaluating whether baseline geometry predicts recurrence, reverse remodeling, symptom improvement, hospitalization, and need for repeat intervention. The ultimate aim is not to add complexity to AF assessment, but to transform 3D geometry into a reproducible marker of AtCM that can support individualized rhythm-control care.

## 8. Conclusions

Three-dimensional LA geometry offers a more comprehensive framework for AF evaluation than linear or volumetric measures alone by capturing chamber shape, regional deformation, PV and LAA anatomy, posterior wall configuration, and wall-thickness heterogeneity. As a macroscopic phenotype of AtCM, it may reflect the combined effects of wall stress, fibrosis, adiposity, mechanical dysfunction, and electrical remodeling. Although geometry-derived markers show prognostic value for AF persistence, thromboembolic substrate, and post-ablation recurrence, methodological heterogeneity and limited prospective validation—especially in the PFA era—currently preclude standalone clinical use. Future standardized, multimodal, and AI-enabled approaches may help translate 3D geometry into individualized rhythm-control selection and post-ablation surveillance.

## Figures and Tables

**Table 1:** Imaging platforms for three-dimensional left atrial reconstruction in atrial fibrillation.

Modality	Strengths	Limitations	Best Use in AF	Primary Evidence
Cardiac CT	High isotropic spatial resolution; excellent definition of the LA body, PV ostia, PV variants, LAA anatomy, posterior wall, left lateral ridge, and extracardiac relationships; compatible with semi-automated segmentation, EAM integration, and quantitative shape analysis.	Ionizing radiation; iodinated contrast exposure; image quality influenced by rhythm irregularity, heart rate, acquisition phase, and segmentation thresholds; primarily anatomical, with limited direct tissue characterization.	Pre-ablation anatomical planning and anatomical digital twinning; PV/LAA roadmapping; extraction of LAV/LAVI, LASP, ASI, AEI, PV geometry, LAA morphology, and surface-based shape descriptors.	Jongbloed et al. [43]; Chen et al. [44]; Kistler et al. [45]; Bisbal et al. [31]
CMR	No ionizing radiation; enables LA/PV anatomical imaging, cine-based functional assessment, strain/volumetric analysis, and LGE-based quantification of fibrosis or post-ablation scar; suitable for integrated anatomical-substrate phenotyping.	Lower spatial resolution than CT for fine PV/LAA anatomy; longer acquisition time; susceptibility to arrhythmia and respiratory motion; limited availability; gadolinium exposure; LGE segmentation remains technically demanding and incompletely standardized.	Substrate characterization; assessment of LA fibrosis, scar, function, and reverse remodeling; complementary phenotyping when anatomical shape is interpreted alongside tissue disease.	Kato et al. [46]; Marrouche et al. [47]; DECAAF II Investigators [48]; Bieging et al. [42]
3D echocardiography	Widely available; repeatable; no radiation or contrast; real-time assessment of LA volume and function; useful for longitudinal follow-up and reverse-remodeling assessment.	Operator- and acoustic-window dependent; lower spatial resolution than CT/CMR; limited visualization of PVs, posterior wall, and LAA; tends to underestimate LA volume compared with CT/CMR; less suitable for detailed surface-based geometry.	Volume and functional assessment; serial evaluation of LAV/LAVI, LA emptying function, and post-ablation remodeling; pragmatic adjunct to CT/CMR-derived anatomical models.	Mor-Avi et al. [49]; Miyasaka et al. [50]; Marsan et al. [51]; Faletra et al. [52]
Electroanatomic mapping	Provides intraprocedural LA geometry combined with voltage, activation timing, fractionation, conduction properties, and low-voltage substrate; high-density systems improve spatial sampling; allows integration with CT/CMR shells.	Invasive; geometry is catheter-sampled and interpolated rather than image-derived; dependent on contact, point density, rhythm during acquisition, loading conditions, respiratory/cardiac motion, electrode configuration, and voltage thresholds.	Voltage-anatomy integration and procedural substrate mapping; identification of low-voltage areas, conduction abnormalities, ablation gaps, and regional electrical substrate during ablation.	Rolf et al. [53]; Jadidi et al. [54]; Vlachos et al. [55]

Abbreviations: AF, atrial fibrillation; AEI, atrial eccentricity index; ASI, atrial asymmetry index; CMR, cardiac magnetic resonance; CT, computed tomography; EAM, electroanatomic mapping; LA, left atrial; LAA, left atrial appendage; LASP, left atrial sphericity; LAV, left atrial volume; LAVI, indexed left atrial volume; LGE, late gadolinium enhancement; PV, pulmonary vein.

**Table 2:** Quantitative Three-Dimensional Atrial Geometry Metrics in Atrial Fibrillation.

Metric/Domain	Technical Definition	Biological Interpretation	Main Clinical Relevance	Key Studies
LAV/LAVI	Total LA volume derived from 3D reconstruction; indexed to body surface area for LAVI.	Global volumetric burden of atrial remodeling; reflects cumulative pressure, volume, metabolic, and arrhythmic stress.	Established predictor of AF persistence, rhythm-control failure, and recurrence after catheter ablation; useful baseline structural marker but lacks regional information.	Njoku et al. [24]; Costa et al. [80]; den Uijl et al. [81]; Marsan et al. [51]; Mor-Avi et al. [49]; Bossard et al. [82]; Matei et al. [83]; Miyasaka et al. [50]
LASP/LA sphericity	Quantifies how closely LA shape approximates or deviates from an ideal sphere; calculated using volume-based, surface-based, or point-cloud methods.	Global shape transformation; may reflect advanced atrial cardiomyopathy, wall-stress adaptation, loss of normal atrial architecture, and impaired mechanical function.	Predicts recurrence after cardioversion and ablation in several cohorts; may improve thromboembolic and atrial-myopathy phenotyping, although results are method-sensitive.	Bisbal et al. [31,33,84]; Nakamori et al. [32]; Bieging et al. [42]; Osmanagic et al. [85]; Shi et al. [86]; Hopman et al. [87]; Dudzińska-Szczerba et al. [88]; Ferkh et al. [89]; Guo et al. [90]; Mulder et al. [91]
ASI	Ratio of anterior LA volume to total LA volume after compartmental segmentation of the LA.	Directional, asymmetric anterior remodeling; reflects nonuniform atrial deformation influenced by extracardiac constraints and chronic filling-pressure burden.	Marker of regional atrial remodeling; associated with AF phenotype, diastolic dysfunction, activation pattern, and long-term ablation outcomes in selected cohorts.	Nedios et al. [13,30,92,93]; Guo et al. [90]
AEI	Ratio of maximum LA diameter to mean LA diameter derived from 3D reconstruction.	Eccentric elongation and anisotropic deformation rather than spherical expansion.	Emerging descriptor that may distinguish elongated from spherical remodeling phenotypes among patients with similar LAV; currently less validated in AF ablation cohorts.	Yi et al. [94]
PV anatomy and orientation	PV number, common trunks, accessory veins, ostial area, ovality, angulation, takeoff orientation, and antral geometry.	Trigger-related anatomy and procedural interface between LA body and PV myocardial sleeves.	Relevant to PVI planning, catheter stability, cryoballoon occlusion, lesion contiguity, PV reconnection, and recurrence risk.	Sorgente et al. [37]; Sohns et al. [95]; Wei et al. [96]; Knecht et al. [97]; Hayashi et al. [98]; Isgandarova et al. [99]; Boussoussou et al. [100]; LaBarbera et al. [101]
LAA morphology and ostial geometry	LAA shape category, ostial size, takeoff height, bend angle, landing-zone geometry, depth, volume, and relation to the left superior PV/lateral ridge.	Appendage-specific component of atrial remodeling; may influence thrombus formation, flow stasis, LAAC feasibility, and recurrence risk.	Relevant to stroke-risk phenotyping, LAAC planning, and selected recurrence models after ablation; quantitative assessment may outperform simple morphology categories.	Di Biase et al. [102]; Nedios et al. [103]; Smit et al. [104]; Bieging et al. [105]; Gong et al. [106]; Simon et al. [36]; Papathanasiou et al. [107]; Liu et al. [108]; Lin et al. [109]
Posterior wall, roof, and lateral ridge geometry	Regional shape, curvature, deformation, flattening, and spatial relationships involving the posterior wall, LA roof, mitral isthmus, and left lateral ridge.	Region-specific substrate architecture; links anatomical deformation with low-voltage substrate, conduction abnormalities, and lesion-delivery challenges.	May identify recurrence-prone anatomical regions not captured by LAV or LASP; relevant to substrate mapping and adjunctive ablation planning.	Kurotobi et al. [110]; Jia et al. [14]; Nedios et al. [111]; Sun et al. [112]
LA wall thickness	Regional LA wall thickness measured by CT or CMR, particularly around PV antra, posterior wall, roof, septum, and left lateral ridge.	Lesion-relevant anatomy; reflects local tissue substrate and may influence transmurality, reconnection, and ablation biophysics.	Potential guide for personalized RF lesion delivery and ablation-index targets; relationship with acute success and recurrence remains heterogeneous.	Beinart et al. [113]; Inoue et al. [114]; Mulder et al. [115]; Teres et al. [116]; Oh et al. [117]; Lee et al. [118]; Boussoussou et al. [100]; Silva Cunha et al. [119]; Liu et al. [120]
Surface curvature, radiomics, fractal and AI-derived descriptors	High-dimensional computational features derived from LA/PV surface geometry, texture, curvature, atlas registration, statistical shape modeling, or machine learning.	Captures complex geometric patterns not reducible to predefined indices; may identify latent anatomical phenotypes of atrial cardiomyopathy.	Emerging approach for recurrence prediction, digital-twin modeling, and automated substrate phenotyping; requires interpretability and external validation.	Varela et al. [121]; Firouznia et al. [122]; Atta-Fosu et al. [123]; Roney et al. [124]; Kim et al. [125]; Razeghi et al. [126]

Abbreviations: AEI, atrial eccentricity index; AF, atrial fibrillation; AI, artificial intelligence; ASI, atrial asymmetry index; CMR, cardiovascular magnetic resonance; CT, computed tomography; LA, left atrial/left atrium; LAA, left atrial appendage; LAAC, left atrial appendage closure; LASP, left atrial sphericity; LAV, left atrial volume; LAVI, left atrial volume index; PV, pulmonary vein; PVI, pulmonary vein isolation; RF, radiofrequency.

**Table 3:** Key rhythm-control and ablation-outcome studies linking 3D atrial geometry, mechanics, and substrate phenotyping with AF ablation outcomes.

Author, Year	Population/Design	Imaging or Computational Approach	Rhythm-Control Endpoint	Main Finding
Jing et al., 2025 [156]	512 patients with AF undergoing first catheter ablation; retrospective CTA-based analysis	CTA-derived LA fractal dimension as a quantitative marker of morphologic heterogeneity	AF recurrence and non-improvement in EHRA symptom score after ablation	Higher LA fractal dimension independently predicted both recurrence and symptom non-improvement; LA-FD >1.208 identified a higher-risk phenotype
Sillett et al., 2025 [157]	Patients with AF undergoing catheter ablation; retrospective gated CT analysis	4D CT-derived 3D LA motion, reservoir strain, contractile strain, and strain-rate parameters	AF phenotype and recurrence after catheter ablation	Reduced passive and active 3D LA motion associated with more advanced AF phenotype and post-ablation recurrence
Chen et al., 2026 [158]	AF patients undergoing catheter ablation; CTA-based regional remodeling analysis	LA posterior volume and posterior/anterior volume ratio	AF recurrence after ablation	Posterior LA remodeling metrics were associated with recurrence risk after ablation
Lin et al., 2025 [109]	463 patients undergoing first-time AF ablation	CT-based LAA morphology classification.	Atrial tachyarrhythmia recurrence within 1 year	Windsock-type LAA morphology was associated with the highest recurrence risk and independently predicted recurrence
Gomes et al., 2025 [79]	439 patients undergoing RF pulmonary vein isolation	Machine-learning-derived CT measurement of LA wall thickness	Long-term AF recurrence after the blanking period	Mean LA wall thickness independently predicted time to AF recurrence after PVI
Alderete et al., 2024 [159]	Prospective multicenter Ablate-by-LAWT study in paroxysmal AF	MDCT-derived LA wall thickness used to adapt ablation index to local atrial thickness	Procedural feasibility, safety, and rhythm outcome after personalized PVI	LAWT-guided ablation was feasible and supported individualized RF energy delivery according to local atrial anatomy
Falasconi et al., 2025 [160]	PeAF-by-LAWT randomized trial in persistent AF	MDCT-derived LA wall thickness to personalize PVI lesion delivery	12-month atrial arrhythmia-free survival and procedural metrics	LAWT-guided PVI was non-inferior to the CLOSE protocol and reduced RF/procedural burden
Invers-Rubio et al., 2024 [161]	AF patients undergoing PVI with non-invasive conduction assessment	Regional conduction velocity mapping using non-invasive electrocardiographic imaging approaches	Arrhythmia-free survival after ablation	Regional conduction velocity features were associated with post-PVI rhythm outcome
Sharp et al., 2025 [162]	22 patients undergoing de novo ablation for persistent AF	LA SSM integrating CDM-identified pivoting/rotational propagation patterns with voltage and conduction mapping	Localization of potential extra-PV ablation targets	CDM propagation patterns clustered in reproducible anteroseptal and inferoposterior LA regions; conventional LVA/CV mapping did not reliably identify these regions
Sharp et al., 2025 [162]	Patients with AF undergoing MRI and global chamber CDM	Integration of MRI-derived LA geometry with global chamber charge-density mapping	Multimodal atrial substrate characterization	Demonstrated technical integration of MRI-derived LA geometry with CDM to support spatially consistent substrate assessment
Bifulco et al., 2025 [163]	Post-ablation AF cohort with clinical and imaging-derived predictors	Explainable machine-learning model incorporating clinical and LGE-MRI substrate variables	Arrhythmia recurrence after ablation	Explainable ML predicted recurrence and identified patient-specific contributors to risk, including imaging substrate features
Zhang et al., 2025 [164]	109 patients with paroxysmal AF undergoing RF catheter ablation	Three-dimensional speckle-tracking echocardiography-derived LA strain	1-year AF recurrence	LA reservoir strain independently predicted AF recurrence; reported cutoff was 16.5%
Du et al., 2026 [165]	130 AF patients undergoing catheter ablation; retrospective CT-based study	Cardiac CT-derived left atrioventricular coupling index; LACI defined as LA/LV diastolic volume ratio	AF recurrence at 18 months	Recurrence occurred in 29.2%; LACI was higher in patients with recurrence and independently predicted recurrence; LACI ≥89% identified lower recurrence-free survival

Abbreviations: AF, atrial fibrillation; ASI, asymmetry index; CDM, charge-density mapping; CT/CTA, computed tomography/computed tomography angiography; CV, conduction velocity; EHRA, European Heart Rhythm Association; LA, left atrium; LAA, left atrial appendage; LASP, left atrial sphericity; LAV, left atrial volume; LAWT, left atrial wall thickness; LGE-MRI, late gadolinium enhancement magnetic resonance imaging; LVA, low-voltage area; MDCT, multidetector computed tomography; PVI, pulmonary vein isolation; RF, radiofrequency; SSM, statistical shape modeling.

## Data Availability

All data generated in this research is included within the article.
